# Supplementary material for: The role of gadolinium in magnetic resonance imaging for early prostate cancer diagnosis: A diagnostic accuracy study
Source: PLoS One. 2019 Dec 23;14(12):e0227031. doi: 10.1371/journal.pone.0227031 (PMC6927639; doi:10.1371/journal.pone.0227031)
Supplement: S1 Table — N/n = patients/lesions. All values are presented as mean/standard deviation. PI-RADS 2 lesions were higher classified and biopsied according to the PI-RADSv1 system, then downgraded to PI-RADS 2 upon PI-RADSv2 re-evaluation. CSPca; Clinically Significant Prostate cancer, IPca; Insignificant Prostate cancer, mpMRI; multiparametric MRI. (DOCX) [file pone.0227031.s001.docx]

# S1 Table. Baseline characteristics of patients and lesions.

| **Variable** | **Value** |
| --- | --- |
| Age (y) | 64±7 |
| Tumor size (min-max, mean±*σ* mm) | 5-57, 14±6 |
| Interval between MRI and biopsy (d) | 40±38 |
| Field strength (N/n) |  |
| 1.5T | 10/15 |
| 3.0T | 203/271 |
| Prostate zone |  |
| Peripheral (All/IPCa/CSPca) | 116/79/48 |
| Transitional (All/IPCa/CSPca) | 170/74/41 |
| Gleason |  |
| 6 | 33 |
| 7 | 64 |
| 8 | 11 |
| 9 | 8 |
| 10 | 2 |
| Clinical nature of cancer |  |
| 1.5T, All/CSPca | 12/3 |
| 3.0T, All/CSPca | 141/83 |
| PI-RADSv2.0 (mpMRI) |  |
| 2 | 3 |
| 3 | 18 |
| 4 | 165 |
| 5 | 84 |

N/n = patients/lesions. All values are presented as mean/standard deviation. PI-RADS 2 lesions were higher classified and biopsied according to the PI-RADSv1 system, then downgraded to PI-RADS 2 upon PI-RADSv2 re-evaluation. CSPca; Clinically Significant Prostate cancer, IPca; Insignificant Prostate cancer, mpMRI; multiparametric MRI
