# Supplementary material for: The role of gadolinium in magnetic resonance imaging for early prostate cancer diagnosis: A diagnostic accuracy study
Source: PLoS One. 2019 Dec 23;14(12):e0227031. doi: 10.1371/journal.pone.0227031 (PMC6927639; doi:10.1371/journal.pone.0227031)
Supplement: S2 Table — T2 TSE, T2-weighted Turbo Spin Echo; DWI, Diffusion-Weighted Imaging; DCE, Dynamic Contrast Enhancement; TE, Echo Time; TR, Repetition Time; FH, Foot-Head direction; RL, Right-Left direction; AP, Anterior-Posterior direction. (DOCX) [file pone.0227031.s002.docx]

**S2 Table. Prostate MRI technical parameters.**

|  | **T2 TSE** | | **DWI** | | **DCE** | |
| --- | --- | --- | --- | --- | --- | --- |
| Patient position | Prone, arms down at sides, head first | | | | | |
| Imaging plane | axial | | | | | |
| Coil | dStream FlexCoverage Anterior Coil (Philips) | | | | | |
| Contrast | No | | No | | Gadoteridol  0.1 mmol/kg | |
| Anatomic coverage | Pelvis | | | | | |
|  | **3T** | **1.5T** | **3T** | **1.5T** | **3T** | **1.5T** |
| TE (ms) | 120 | 100 | 60 | 66 | 1.91 | 2.8 |
| TR (ms) | 4530 | 4530 | 6253 | 3585 | 5.50 | 1.50 |
| Flip angle ^o^ | 90 | 90 | 90 | 90 | 15 | 10 |
| TSE factor | 25 | 23 |  |  |  |  |
| Number of slices | 25 | 15 | 25 | 40 | 40 | 30 |
| Acquisition voxel size  RL×AP (mm) | 0.5×0.5 | 0.55×0.55 | 2.00×2.03 | 2.19×2.45 | 1.40×1.02 | 1.55×1.57 |
| Recon voxel size AP×RL (mm) | 0.31×0.31 | 0.27×0.27 | 2×2 | 1.25×1.25 | 1.02×1.02 | 0.91×0.91 |
| Slice thickness (mm) | 3 | 4 | 3 | 3 | 3 | 3 |
| Spacing (mm) | 0 | 0.4 | 0 | 0 |  |  |
| FOV FH×RL×AP (mm) | 75×160  ×160 | 65×140  ×140 | 75×256  ×256 | 120×140  ×140 | 120×262  ×262 | 120×262  ×262 |
| Acquisition matrix (frequency× phase) | 320×312 | 256×245 | 128×124 | 64×57 | 256×186 | 168×166 |
| Acquisition time (min) | 3:55 | 3:54 | 5:56 | 5:31 | 5:31 | 6:12 |

T2 TSE, T2-weighted Turbo Spin Echo; DWI, Diffusion-Weighted Imaging; DCE, Dynamic Contrast Enhancement; TE, Echo Time; TR, Repetition Time; FH, Foot-Head direction; RL, Right-Left direction; AP, Anterior-Posterior direction
