# Supplementary material for: The role of gadolinium in magnetic resonance imaging for early prostate cancer diagnosis: A diagnostic accuracy study
Source: PLoS One. 2019 Dec 23;14(12):e0227031. doi: 10.1371/journal.pone.0227031 (PMC6927639; doi:10.1371/journal.pone.0227031)
Supplement: S3 Table — (DOCX) [file pone.0227031.s003.docx]

**S3 Table. The character of lesions overcalled by the dynamic contrast enhancement.**

| **Dignity** | **Transitional zone** | | **Peripheral zone** | |
| --- | --- | --- | --- | --- |
|  | *All (n)* | *Overcall (n)/%* | *All (n)* | *Overcall (n)/%* |
| Prostatitis | 46 | 32/70 | 13 | 9/69 |
| Benign prostate hyperplasia | 25 | 15/60 | 14 | 8/57 |
| ASAP | 17 | 13/77 | 14 | 12/86 |
| Gleason 3+3 | 16 | 12/75 | 17 | 11/65 |
| Prostate tissue without pathology | 23 | 15/65 | 8 | 5/63 |
| Periprostatic tissue | 0 | 0/0 | 2 | 0/0 |
