# Supplementary material for: The role of gadolinium in magnetic resonance imaging for early prostate cancer diagnosis: A diagnostic accuracy study
Source: PLoS One. 2019 Dec 23;14(12):e0227031. doi: 10.1371/journal.pone.0227031 (PMC6927639; doi:10.1371/journal.pone.0227031)
Supplement: Attachment — Submitted filename: PLOSoneR01.docx [file pone.0227031.s007.docx]

Response to the reviewer(s)

Reviewer #1: Congratulations to this overall well-written manuscript, which confirms the results of several prior studies in the field.

Re: We sincerely thank the reviewer for the time spent on our manuscript and for the relevant comments that improve the manuscript´s readability. We were glad to cover the requested issues in the following point-by-point response.

001-MR-guided biopsy: Was an additional systematic biopsy performed (TRUS, transperineal?).

Re-001: Indeed, most of the patients included in this study were referred to our department upon persisting suspicion for malignancy after a negative ambulant TRUS-biopsy. Rare exceptions count for less than 10% of the available dataset. This selection line reflects on the disproportionally large number of Tz and small lesions, which are less likely to allocate without targeting. After an MRI-guided prostate biopsy, no further TRUS was performed. In N= 6 cases, however, the MRI-guided biopsy was repeated within a time interval of 612±231 (mean, std) days months because of a persisting elevation of the PSA. The treatment decision was based on the second biopsy.

The methods’ part was updated accordingly (L120-127).

002- Additionally to the Gleason score, is it possible for the authors to provide data on tumor volume estimates? (either by MR segmentation or biopsy core length)

Re-002: We thank the reviewer for this relevant comment and the opportunity to discuss how the tumor volume might be connected to the DCE-influence of the PIRADS predictive value. We upgraded Figure 3 and added histograms of the tumor size for each prostate zone separately (Fig. 3 I, J). The lesion size was measured as the maximal diameter in paraxial T2w sections.

Moreover, the reviewer´s comment motivated us to analyze the impact of DCE for different tumor sizes in the Pz and Tz, setting 11 mm as an arbitrary limit for “small” and “large” lesions (Figure 3 C, D, G, H). Interestingly, the addition of DCE significantly worsens the PIRADS prognostic value for small Tz tumors. In the other groups, the effect remains equivocal. The main text and Figure 3 were updated respectively (L319-L326 and Fig. 3 files and caption).

003- Readers: Please provide initials of the two readers (or names if not one of the authors) and years since board certification (or years in training)

Re-003: The initials and experience of the readers were added in text, L143-144.

004- Please provide exact p-values instead of dichotomized >/< 0.05 in the results section.

Re-004: The exact p-values were provided the results section, L319 and L326

005- Consider omitting "Another critical point is the lack of healthy subjects since only patients with a level of suspicion were screened and biopsied." I fail to see how this is a limitation.

Re-005: We thank the reviewer for this comment. The relevant text was removed (L414-415)

006- Another point that might add to the discussion is the simplification of DCE from PIRADS v1 to v2 and studies showing increased interreader agreement. Might biparametric MRI further improve interreader agreement?
Re-006: The reviewer raises a crucial point for the clinical implementation of PIRADS – the interrater agreement. PIRADSv1 was a powerful albeit complex scoring system, leading to an interrater agreement rate of 0.39 – 0.64 (Becker et al., 2017; Tewes et al., 2016; Krishna et al., 2017). Simplification of the scoring system in PIRADSv2 significantly improved the interrater agreement: from 0.64 to 0.70 in the study of Becker et al., from 0.39 to 0.56 in the study of Tewes et al. and from 1.33 (Bland-Altman statistics for sum-score) to 0.41 in Krishna et al. Interobserver agreement studies for mp- vs. bpMRI were not performed yet, a further simplification is nevertheless quite likely to improve the interrater agreement. Krishna et al. show that the kappa rate of DWI for the peripheral zone (equaling PIRADSv2 without DCE) was 0.51, improved compared to the PIRADSv2 kappa = 0.41. This highlights the necessity for dedicated study designs for an unbiased, experience-weighted (Gatti et al. 2019) evaluation of the interrater performance between mp- and bpMRI.

The discussion was upgraded accordingly (L 401-411)

007- Consider adding CIs to Fig 3. Otherwise, the figures are very nice and appealing.

Re-007: CI at 95% were added in the legend of Figure 3, L328-343

008- I applaud the authors for providing the data on a granular, per-lesion level. Although in the interest of a short review turnover time, I have not verified the results myself, this will certainly help interested readers to do so, and other researchers when accruing data for meta-analyses.

Re-008: We are pleased to read the reviewer´s opinion, that the supplementation of the original dataset is a *sine-qua-non* for qualitative scientific communication. We are grateful to the editor for supplying a data-exchange platform. We will be glad to support any further implementation of our dataset in a meta-analysis or other systematic review format.
